# Expression and purification of E140 protein antigen fragments of *Plasmodium vivax* and *Plasmodium berghei* for serological assays

**DOI:** 10.1002/2211-5463.13939

**Published:** 2025-01-15

**Authors:** Rodolfo Ferreira Marques, Edit Ábrahám, Hiromi Muramatsu, Daniel Youssef Bargieri, Norbert Pardi, Zoltán Lipinszki

**Affiliations:** ^1^ Department of Microbiology Perelman School of Medicine, University of Pennsylvania Philadelphia PA USA; ^2^ Department of Parasitology Institute of Biomedical Sciences, University of Sao Paulo São Paulo Brazil; ^3^ Synthetic and Systems Biology Unit Institute of Biochemistry, HUN‐REN Biological Research Centre Szeged Hungary; ^4^ National Laboratory for Biotechnology Institute of Genetics, HUN‐REN Biological Research Centre Szeged Hungary; ^5^ ATGandCo Biotechnology Ltd Mórahalom Hungary

**Keywords:** ELISA, Malaria, mRNA‐lipid nanoparticle, *Plasmodium* spp., protein purification, recombinant E140 fragments, serological assays, vaccine development

## Abstract

Malaria, a life‐threatening disease caused by *Plasmodium* parasites, continues to pose a significant global health threat, with nearly 250 million infections and over 600 000 deaths reported annually by the WHO. Fighting malaria is particularly challenging partly due to the complex life cycle of the parasite. However, technological breakthroughs such as the development of the nucleoside‐modified mRNA lipid nanoparticle (mRNA‐LNP) vaccine platform, along with the discovery of novel conserved *Plasmodium* antigens such as the E140 protein, present new opportunities in malaria prevention. Importantly, production of recombinant proteins for malaria vaccine evaluation by serological assays often represents an additional hurdle because many *Plasmodium* proteins are complex and often contain transmembrane domains that make production and purification particularly difficult. This research protocol provides a step‐by‐step guide for the production and purification of *P. berghei* and *P. vivax* E140 protein fragments that can be used to test humoral immune responses against this novel malaria vaccine target. We demonstrate that the purified proteins can be successfully used in enzyme‐linked immunosorbent assay (ELISA) to evaluate antigen‐specific binding antibody responses in sera obtained from E140 mRNA‐LNP‐vaccinated mice. Therefore, these proteins can contribute to the development and evaluation of E140‐based malaria vaccines.

## Graphical abstract

We provide a step‐by‐step guide for producing E140 antigen fragments from *Plasmodium berghei* (Pb1) and *Plasmodium vivax* (Pv1). Pb1/Pv1 are expressed in *E. coli*, solubilized by freeze–thawing, refolded by slow dilution, purified by affinity chromatography (IMAC), then concentrated and subjected to quality control. The intact proteins can be used in immunological assays (ELISA) to test antibody‐containing samples from vaccinated mice.

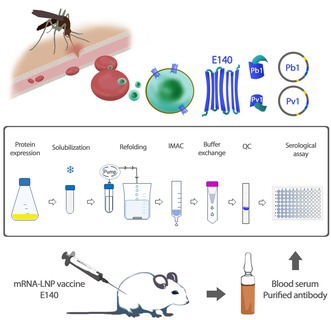

**Abbreviations**
aa: amino acid
DNA: deoxyribonucleic acid
*E. coli*
: *Escherichia coli*
ELISA: enzyme‐linked immunosorbent assay
ELISpot: enzyme‐linked immunosorbent spot
h: hour(s)
IMAC: immobilized‐metal affinity chromatography
IPTG: isopropyl‐beta‐D‐thiogalactopyranoside
kDa: kilodalton
L: liter
LB: Luria‐Bertani medium
LNP: lipid nanoparticle
max: maximum
min: minute
mL: milliliter
mRNA: messenger ribonucleic acid
MW: molecular weight
MWCO: molecular weight cut‐off
PBS: phosphate‐buffered saline
PES: polyethersulfone
RT: room temperature
s: seconds
SDS‐PAGE: sodium dodecyl sulfate‐polyacrylamide gel electrophoresis
TBS: Tris‐buffered saline
ε: molecular extinction coefficient
μL: microliter

Malaria remains a serious public health challenge and is the most devastating tropical parasitic disease globally. In 2022, approximately 249 million cases of malaria were recorded, resulting in 608 000 deaths worldwide. Since 2000, the incidence of malaria in at‐risk areas has decreased from 80 to 58 cases per 1000 individuals. However, this trend does not hold true for the Americas, where an increase in malaria cases is recorded [1]. In the Amazon basin, malaria primarily affects the local population, accounting for 75% of cases in the Americas. In Brazil, *Plasmodium vivax* is the most prevalent causative agent of malaria, responsible for 86% of cases registered in 2021 [1]. These statistics underscore the critical need for worldwide prevention and control of malaria, particularly in high‐incidence regions [1].

The primary strategies to combat malaria include vector control, rapid diagnosis and treatment, and vaccine development [2]. However, developing a safe and efficient vaccine has proven particularly challenging due to the biological complexity of the *Plasmodium* parasite, which has a complex life cycle encompassing both asexual and sexual stages, and significant genetic diversity that enables the parasite to evade the host's immune response. Currently, vaccine approaches employ various platform technologies, including recombinant proteins, viral vectors, virus‐like particles, plasmid DNA, mRNA, synthetic peptides, and irradiated or attenuated sporozoites [3].

The mRNA vaccine technology holds great promise for developing effective malaria vaccines [4], as it has been shown to induce robust protective cellular and humoral immune responses, as demonstrated during the SARS‐CoV‐2 pandemic [5]. Given the potential to develop multivalent mRNA vaccines [6, 7], it is possible to design vaccines targeting multiple stages of the *Plasmodium* life cycle, potentially enhancing their effectiveness by blocking the infection in different stages.

A major challenge of malaria vaccine research is the identification of new effective antigens. Recently, Smith *et al*. described a promising novel antigen, E140, which is expressed in multiple stages of the *Plasmodium* lifecycle, including sporozoites, liver stages, and blood stages [8]. It is localized at the apical and posterior ends of sporozoites, on the parasitophorous vacuole membrane during the liver stage, and in developing merozoites during the blood stage, making it a promising vaccine target. In challenge studies using *Plasmodium yoelii* (Py) in murine infection models, vaccination with PyE140‐encoding DNA using HuAd5 vector, induced sterilizing protection in up to 100% of immunized animals. Orthologs of the *E140* are present across all known *Plasmodium* species, with high sequence similarity between them, suggesting that E140 could be an effective vaccine antigen against *Plasmodium*.

The evaluation of an E140‐based vaccine requires the production of recombinant protein for serological assays. However, producing these proteins in heterologous systems is challenging due to their membrane association, which is mediated by their five predicted transmembrane domains [8] (Fig. 1A). To address this issue, we used DeepTMHMM [9] to determine the transmembrane regions of the *P. berghei* E140 (rodent malaria model, PbE140) and the *P. vivax* E140 (PvE140), selecting two fragments from each protein (Pb1 and Pb2, and Pv1 and Pv2, Fig. 1A) that are external to the plasma membrane—a common strategy for producing *Plasmodium* antigen fragments for serological tests [10]. These antigens were expressed in *E. coli*, forming inclusion bodies. We found that a modified version of a freeze/thaw solubilization technique [11] followed by dilution‐based refolding [12] and affinity‐purification [13] yielded the best quality of soluble recombinant protein antigens (Fig. 2).

**Fig. 1:**
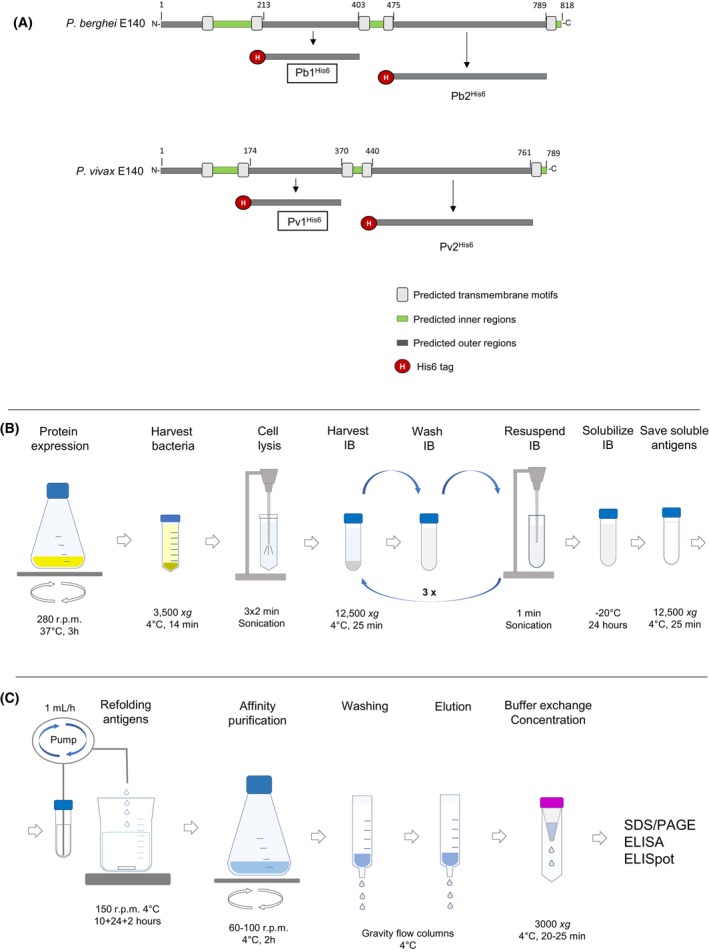
Expression and multistep purification of recombinant fragments of the *Plasmodium* E140 protein antigen from *E. coli* cells. (A) Schematic representation of *Plasmodium berghei* E140 (top) and *Plasmodium vivax* E140 (bottom) antigens as predicted by DeepTMHMM [9]. External protein regions (dark gray bars) were cloned and expressed as recombinant proteins (designated as Pb1, Pb2, Pv1, and Pv2). This protocol describes the production of Pb1^His6^ and Pv1^His6^ (indicated by black frames) and their use in ELISA. Numbers on the diagrams correspond to amino acid positions. (B) Illustration of the process for expressing Pb1^His6^ and Pv1^His6^ in *E. coli* cells, including inclusion body (IB) preparation, washing, and antigen solubilization. (C) Diagram depicting the steps involved in antigen refolding, affinity purification by IMAC, buffer exchange, and concentration. ELISA, enzyme‐linked immunosorbent assay; ELISpot, enzyme‐linked immunosorbent spot; IMAC, immobilized metal affinity chromatography; SDS‐PAGE, sodium dodecyl sulfate‐polyacrylamide gel electrophoresis.

**Fig. 2:**
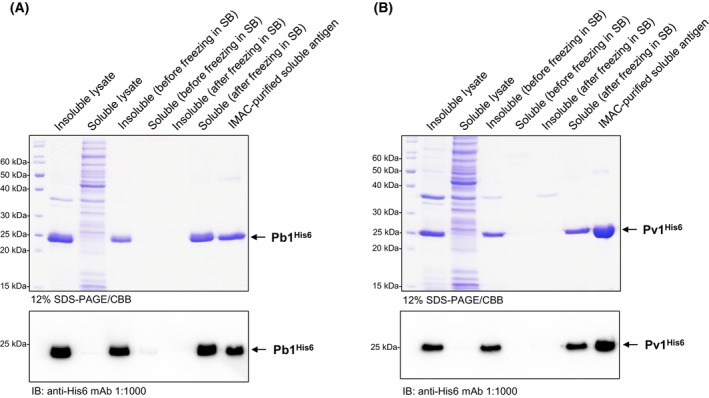
The multistep purification process of recombinant Pb1^His6^ and Pv1^His6^ yields soluble and pure protein antigens. Coomassie Brilliant Blue‐stained (CBB) 12% SDS‐PAGE gels (top panels) and immunoblots (bottom panels) using anti‐His6 monoclonal antibody (#MA121315, Thermo Fisher Scientific, Waltham, MA, USA) demonstrate the solubility and purity of (A) Pb1^His6^ and (B) Pv1^His6^ recombinant fragments of the *Plasmodium* E140 antigen. Most of the recombinant proteins are initially insoluble in the bacterial lysate and remain insoluble after inclusion body resuspension in solubilizing buffer (SB). However, after a freeze‐thaw cycle in SB, both proteins become soluble, and can be refolded and affinity‐purified to homogeneity.

In this research protocol, we provide a detailed, step‐by‐step guide for the production, solubilization, refolding, and purification of Pb1 and Pv1 E140 protein antigen fragments in *E. coli* cells (Fig. 1B, C), with a yield of ~2 mg per 50 mL of culture. These fragments are expressed, purified, and refolded more effectively than the Pb2 or Pv2 E140 protein fragments, which is why this protocol focuses exclusively on their production. We demonstrate that the Pb1 and Pv1 antigens can be utilized in enzyme‐linked immunosorbent assays (ELISA) to evaluate E140‐specific binding antibody responses in sera obtained from E140 mRNA‐LNP‐immunized mice, facilitating a more comprehensive evaluation of the E140 protein as a vaccine candidate (Fig. 3). In addition, we believe that the purified E140 recombinant protein fragments can also be used to assess responses induced by other vaccination platforms or potentially, for immunization studies, making them a valuable tool for other researchers in the ongoing fight against malaria.

**Fig. 3:**
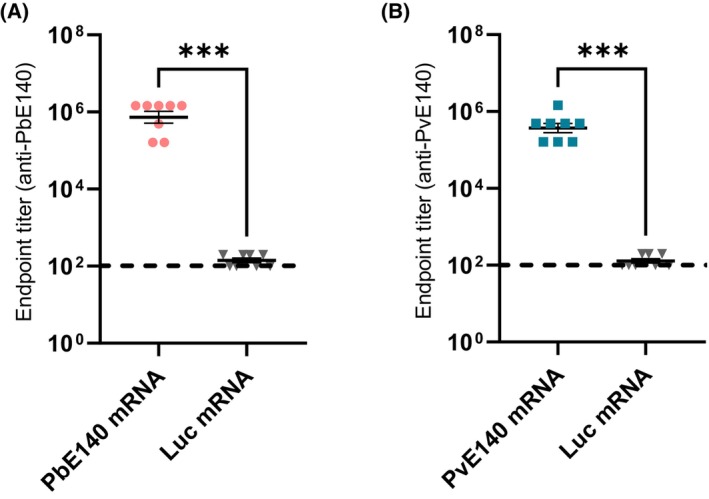
Detection of binding antibody responses in sera obtained from nucleoside‐modified PbE140 mRNA‐LNP‐ or PvE140 mRNA‐LNP‐vaccinated mice by ELISA. Mouse immunizations, ELISA and nucleoside‐modified mRNA‐LNP vaccine production were conducted as described [15, 16]. Eight‐week‐old female BALB/c mice were immunized three times (4 weeks apart) with 5 μg PbE140 or PvE140 mRNA‐LNP vaccines and bled 4 weeks after the last injection. IgG endpoint titers specific to the PbE140 (A) or PvE140 (B) proteins were compared to those obtained from mice immunized with a firefly luciferase (Luc)‐encoding control mRNA‐LNP vaccine. ELISA plates were coated with 100 ng·well^−1^ of purified, soluble recombinant Pb1^His6^ or Pv1^His6^, respectively. Each symbol represents one animal, data is shown as mean ± SEM (*n* = 8 mice per group). The limit of detection is shown as a horizontal dashed line on the graph. Results are expressed as the geometric mean titers in log10 ± SEM and the data were statistically analyzed using a non‐parametric Mann–Whitney test for comparisons between groups. Significant differences between groups are indicated on the graph as ***P < 0.001.

## Materials

### I. Plasmid DNA

Codon‐optimized DNA sequences encoding the 213‐403 amino acid region (designated as Pb1) of the *Plasmodium berghei* E140 protein (UniProt ID: A0A509ADM3) and the 174–370 amino acid region (designated as Pv1) of the *Plasmodium vivax* E140 protein (UniProt ID: A0A564ZQP0) were synthesized by GenScript and cloned into the pET‐DUET‐1 bacterial expression vector (#71146‐3; Merck Millipore, Burlington, MA, USA). Both include an N‐terminal hexa‐histidine affinity tag (His6) for protein purification (Fig. 1A and Tables 1, 2).

**Table 1:** Amino acid sequences of Pb1^His6^ and Pv1^His6^ recombinant fragments of the *Plasmodium berghei* and *Plasmodium vivax* E140 antigen. The N‐terminal hexa histidine tag (His6) is bold.

>Pb1^His6^ *MGSS* **HHHHHH** *SQDPDTQKGINMNICGLSKTVEQFLIDKCPDTKNVNPQCYSLEHAITDAVSVINQYQLMKEFVKKKTNLNKDKGFPIILKYQTGFKMLAKLRDNIDKNVKKLENGYLHTYPILKKLKITLEEVIAKGDDLLNQAESIIDSSKEEVGKIFSNVDNAIINTVYNNVPSLSLKISKLGMYIKKHDENLKIRYVLNKFT* >Pv1^His6^ MGSS**HHHHHH**SQDPSFFKTRNGIYMNVCSASTSIENFLTDRCSVQGGEVDSSCYSLEHIVSDAVSIVEQYQDIKLQIKADLLVDEDRTVPLLTGFLTVLENLKKLQQNVARNNHILEEQYFHTYPVLTRLGRALDAVIQEGEANLQQATGTLDEAKQGVKGAFEEIDQVLGATFKENMEKVNDKITLFNKSINRIIHQYKIKQNLKKYTIS

**Table 2:** Nucleotide sequences of the Pb1^His6^ and Pv1^His6^ recombinant protein‐encoding DNA. The 5′ hexa histidine tag (His6) is bold.

>Pb1^His6^ ATGGGCAGCAGC**CATCACCATCATCACCAC**AGCCAGGATCCtGACACCCAGAAGGGCATCAACATGAACATCTGCGGCCTGTCCAAGACCGTGGAGCAGTTCCTGATCGACAAGTGCCCCGACACCAAGAACGTGAACCCCCAGTGCTACTCCCTGGAGCACGCCATCACCGACGCCGTGTCCGTGATCAACCAGTACCAGCTGATGAAGGAGTTCGTGAAGAAGAAGACCAACCTGAACAAGGACAAGGGCTTCCCCATCATCCTGAAGTACCAGACCGGCTTCAAGATGCTGGCCAAGCTGCGCGACAACATCGACAAGAACGTGAAGAAGCTGGAGAACGGCTACCTGCACACCTACCCCATCCTGAAGAAGCTGAAGATCACCCTGGAGGAGGTGATCGCCAAGGGCGACGACCTGCTGAACCAGGCCGAGTCCATCATCGACTCCTCCAAGGAGGAGGTGGGCAAGATCTTCTCCAACGTGGACAACGCCATCATCAACACCGTGTACAACAACGTGCCCTCCCTGTCCCTGAAGATCTCCAAGCTGGGCATGTACATCAAGAAGCACGACGAGAACCTGAAGATCCGCTACGTGCTGAACAAGTTCACCtAA >Pv1^His6^ ATGGGCAGCAGC**CATCACCATCATCACCAC**AGCCAGGATCCtTCCTTCTTCAAGACCCGCAACGGCATCTACATGAACGTGTGCTCCGCCTCCACCTCCATCGAGAACTTCCTGACCGACCGCTGCTCCGTGCAGGGCGGCGAGGTGGACTCCTCCTGCTACTCCCTGGAGCACATCGTGTCCGACGCCGTGTCCATCGTGGAGCAGTACCAGGACATCAAGCTGCAGATCAAGGCCGACCTGCTGGTGGACGAGGACCGCACCGTGCCCCTGCTGACCGGCTTCCTGACCGTGCTGGAGAACCTGAAGAAGCTGCAGCAGAACGTGGCCCGCAACAACCACATCCTGGAGGAGCAGTACTTCCACACCTACCCCGTGCTGACCCGCCTGGGCCGCGCCCTGGACGCCGTGATCCAGGAGGGCGAGGCCAACCTGCAGCAGGCCACCGGCACCCTGGACGAGGCCAAGCAGGGCGTGAAGGGCGCCTTCGAGGAGATCGACCAGGTGCTGGGCGCCACCTTCAAGGAGAACATGGAGAAGGTGAACGACAAGATCACCCTGTTCAACAAGTCCATCAACCGCATCATCCACCAGTACAAGATCAAGCAGAACCTGAAGAAGTACACCATCTCCtAA

### II. Protein expression

Use sterile glass‐ and plasticware!

1. Chemically competent SixPack [14] *E. coli* cells (Tips & Tricks 1).
2. Luria‐Bertani medium (LB broth): 10 g tryptone, 5 g NaCl, 5 g yeast extract in 1 L ddH_2_O, pH 7.3 (autoclave for 15 min at 120 °C) (Tips & Tricks 2).
3. Carbenicillin·Na_2_‐salt (#15875.03; Serva, Heidelberg, Germany).
4. IPTG (#R0392; Thermo Fisher Scientific, Waltham, MA, USA).
5. Erlenmeyer flasks (300 and 500 mL).
6. 50 mL conical tubes.
7. Thermoregulated laboratory shaker.
8. Refrigerated centrifuge with swing‐out rotor.

### III. Protein purification, buffer exchange, and concentration

Use sterile glass‐ and plasticware!

1. Buffers and stock solution (Tips & Tricks 3):
  1.1 Phosphate‐buffered saline (PBS): 137 mm NaCl, 2.6 mm KCl, 8.7 mm Na₂HPO₄, 1.7 mm NaH₂PO₄. Autoclave and store at 4 °C.
  1.2 Tris‐buffered saline (TBS): 50 mm Tris pH 8.0, 150 mm NaCl. Autoclave and store at 4 °C.
  1.3 Lysis buffer (LyB): TBS, 5 mm 2‐Mercaptoethanol, 1 mm phenylmethylsulfonyl fluoride (PMSF). Prepare fresh.
  1.4 Washing buffer 1 (WB‐1): 50 mm Tris pH 8.0, 300 mm NaCl, 1 mm EDTA, 1% Triton X‐100, 1 m Urea, 5 mm 2‐Mercaptoethanol. Prepare fresh, filter‐sterilize, and store at 4 °C.
  1.5 Washing buffer 2 (WB‐2): TBS, 5 mm 2‐Mercaptoethanol. Prepare fresh.
  1.6 Washing buffer 3 (WB‐3): 50 mm Tris pH 8.0, 500 mm NaCl, 5 mm imidazole. Filter‐sterilize and store at 4 °C.
  1.7 Washing buffer 4 (WB‐4): 50 mm Tris pH 8.0, 500 mm NaCl, 40 mm imidazole. Filter‐sterilize and store at 4 °C.
  1.8 Elution buffer (EB): 50 mm Tris, pH 8.0, 250 mm NaCl, 250 mm imidazole. Filter‐sterilize and store at 4 °C.
  1.9 Solubilizing buffer (SB): TBS, 5 mm 2‐Mercaptoethanol, 2 m Urea. Prepare fresh, filter‐sterilize, and keep at 4 °C.
  1.10 Refolding buffer (RB): 50 mm Tris pH 8.5, 0.4 m sucrose, 10% glycerol, 0.5% Triton X‐100, 0.3 mm GSSG (oxidized glutathione), 3 mm GSH (reduced glutathione). Prepare fresh, filter‐sterilize, and keep at 4 °C.
  1.11 5 m NaCl stock solution: Dissolve 58.44 g of NaCl in 200 mL of ultra‐pure water and autoclave for 15 min at 120 °C. Store at 4 °C.
  1.12 1 m imidazole stock solution: Dissolve 3.4 g of imidazole in 50 mL of ultra‐pure water, filter‐sterilize, and store at room temperature.
2. Microcentrifuge‐, conical‐, and 30 mL Oak Ridge‐type high‐speed tubes.
3. Graduated 10 mL pipettes and pipette aid.
4. Middle and high‐speed refrigerated centrifuges with fixed angle rotors.
5. Sonifier disruptor.
6. Peristaltic pump suitable for small speed applications.
7. Magnetic stirrer and magnetic bars.
8. Glass beakers and flasks.
9. Ni Sepharose 6 Fast Flow resin for IMAC purification (#17531802; Cytiva, Marlborough, MA, USA) (Tips & Tricks 4).
10. Polypropylene empty PD‐10 chromatographic columns (#17043501; Cytiva, Marlborough, MA, USA) or similar.
11. Chromatography column stand.
12. Amicon Ultra‐4 Centrifugal Filter Unit with a 10 kDa MWCO (#UFC801008; Merck Millipore, Burlington, MA, USA).
13. Syringe filters with a cellulose acetate membrane (0.22 μm) and 250–1000 mL vacuum filter units with a polyethersulfone (PES) membrane (0.22 μm) for sterile filtration.
14. 1 mL Luer‐lock syringe.
15. Liquid nitrogen.
16. Spectrophotometer.

## Methods

### I. Expression of recombinant Pb1^His6^ and Pv1^His6^ antigens

Perform all steps at 4 °C unless otherwise specified (Fig. 1B).

1. Transform chemically competent SixPack *E. coli* cells with plasmids encoding Pb1^His6^ and Pv1^His6^ using standard protocols. Plate the transformed cells onto LB agar plates supplemented with 100 μg·mL^−1^ carbenicillin and incubate at 37 °C for 16–18 h.
2. Inoculate 3–5 well‐isolated colonies from the plate into 25 mL of LB broth (supplemented with 100 μg·mL^−1^ carbenicillin) in a 125 mL flask. Incubate at 37 °C with shaking at 280 r.p.m. for 16–18 h.
3. Inoculate 50 mL of LB broth (supplemented with 100 μg·mL^−1^ carbenicillin) in a 300 mL flask with 500 μL of the starter culture.
4. Incubate the culture at 37 °C with shaking until the optical density at 600 nm (A600 nm) reaches 0.4–0.6 (Tips & Tricks 5).
5. Induce protein expression by adding 1 mm IPTG to the culture and grow bacteria at 37 °C with shaking at 280 r.p.m. for 3 h.
6. Place the flask on ice for 10 min, then harvest the cells by centrifugation at 3500 ***g*** for 15 min at 4 °C. Discard the medium.
7. Resuspend the paste in ice‐cold PBS, then centrifuge at 3500 ***g*** for 15 min at 4 °C. Discard the supernatant. Freeze the paste or proceed to Step II/1.

### II. Isolation and solubilization of inclusion bodies

Perform all steps at 4 °C unless otherwise specified (Fig. 1B).

1. Resuspend bacteria in 10 mL lysis buffer (LyB) and lyse the cells by sonication for 2 min (20 s pulses, 30 s breaks, 20% power) on ice. Repeat the sonication twice (Tips & Tricks 6).
2. Centrifuge the lysate at 12 500 ***g*** for 25 min. Decant and discard the supernatant, retaining the insoluble pellet containing the inclusion bodies (IBs, Fig. 2).
3. Resuspend the insoluble pellet thoroughly in 10 mL washing buffer‐1 (WB‐1) using a 10 mL glass pipette, add another 20 mL of WB‐1 and sonicate for 1 min (20 s pulses, 30 s breaks, 10% power) on ice. Centrifuge at 12 500 ***g*** for 25 min. Decant and discard the supernatant, retaining the pellet containing IBs.
4. Repeat Step 3 using 10 + 20 mL of WB‐1 buffer twice.
5. Repeat Step 3 using 10 + 20 mL of washing buffer‐2 (WB‐2).
6. Resuspend the washed IBs in 10 mL of solubilizing buffer (SB) and incubate at 4 °C for 25 min with continuous rotation (20 r.p.m.). Then, transfer the samples into a −20 °C freezer and incubate for 24 h.
7. Thaw the samples at room temperature for 1 h, mix several times, then centrifuge at 12 500 ***g*** for 25 min. Save the supernatant containing the solubilized antigens (Fig. 2) (Tips & Tricks 7).

### III. Refolding the antigens

Perform all steps at 4 °C unless otherwise specified (Fig. 1C)

1. Slowly (flow rate ~ 1 mL·h^−1^) add the solubilized antigens (10 mL each) from Step II/7 to at least 200 mL of refolding buffer (RB) using a peristaltic pump. Mix the buffer/protein solution with a magnetic stirrer at 150 r.p.m.
2. After adding all solubilized proteins to the RB buffer, continue stirring the solution for 24 h to allow complete protein refolding.
3. Gradually supplement the stirring solution with 500 mm NaCl and 5 mm imidazole, adding drop by drop at a slow pace. Stir for another 2 h.
4. Centrifuge the solution at 9000 ***g*** for 25 min. Save the supernatant containing refolded protein antigens (Fig. 2) and transfer it to a prechilled sterile 500 mL flask.

### IV. IMAC‐affinity purification of the antigens

Perform all steps at 4 °C unless otherwise specified (Fig. 1C)

1. Thoroughly mix and homogenize the Ni Sepharose 6 Fast Flow resin. Measure out 2 mL of slurry (equivalent to 50% bed volume) and mix with 20 mL of washing buffer 3 (WB‐3) in a 50 mL conical tube to equilibrate the resin.
2. Centrifuge the tube at 800 ***g*** for 5 min in a swinging‐bucket rotor with low deceleration (e.g., set the brake to 7 out of 10) to prevent turbulence.
3. Carefully decant and discard the supernatant. Resuspend the resin in 10 mL of WB‐3 (Tips & Tricks 8).
4. Transfer the equilibrated resin into a 500 mL Erlenmeyer flask containing the refolded protein antigens from Step III/4.
5. Incubate the flask on an orbital shaker at 60–100 r.p.m. for 2 h.
6. Secure an empty PD‐10 column to a stand.
7. Gradually transfer the resin/sample suspension from the flask to the column, allowing the solution to flow through the resin. Discard the flow‐through.
8. Wash the resin in the column with 4 × 10 mL of WB‐3 buffer, letting the buffer flow through the resin each time. Discard the washing buffer.
9. Wash the resin in the column with 4 × 10 mL of washing buffer‐4 (WB‐4), allowing the buffer to flow through the resin. Discard the flow‐through.
10. Elute the antigens by 4 × 1 mL of elution buffer (EB) in steps into a sterile, pre‐chilled 5 mL tube.

### V. Buffer exchange, concentration, and storage of the antigens

Perform all steps at 4 °C unless otherwise specified (Fig. 1C)

1. Add 5 mL of sterile TBS into a 5 mL centrifugal filter device (concentrator) with a 10 kDa molecular weight cutoff (MWCO). Centrifuge at 4000 ***g*** for 5 min in a swinging‐bucket rotor. Discard the flow‐through.
2. Transfer the eluted proteins from Step IV/10 into the filter device and centrifuge at 3000 ***g*** for 15–25 min until the sample is concentrated to approximately 1 mL. Discard the flow‐through.
3. Add 4 mL of sterile TBS to the concentrated sample. Close the lid, mix by gentle inversion several times, and centrifuge at 3000 ***g*** for 15–25 min until the sample is concentrated to 1 mL. Discard the flow‐through.
4. Repeat Step V/3 five times (Tips & Tricks 9).
5. Collect the buffer‐exchanged and concentrated protein sample from the device (~1 mL) and transfer it to a prechilled tube.
6. Centrifuge the sample at 12 000 ***g*** for 10 min to remove any precipitates formed during buffer exchange and concentration. Save the supernatant and transfer it to a new prechilled tube.
7. Measure the concentration of the purified antigens at 280 nm using a spectrophotometer:
  7.1 Set the molecular weight (MW) to 23.442 kDa for Pb1^His6^, and 23.920 kDa for Pv1^His6^.
  7.2 Set the extinction coefficient (ε) to 11.92 m ^−1^ cm^−1^ (ε/1000) for Pb1^His6^, and 10.43 m ^−1^ cm^−1^ (ε/1000) for Pv1^His6^.
  7.3 If available, set the baseline correction to 340 nm.
  7.4 Use TBS as the blank.
  7.5 Measure the concentration of the purified antigens.
8. Carefully adjust the concentration to 1–2 mg·mL^−1^ with sterile TBS. Test the integrity of the protein by SDS‐PAGE (Fig. 2, last lanes).
9. Filter‐sterilize the purified proteins (optional).
10. Prepare small aliquots as desired.
11. Flash‐freeze the aliquots in liquid nitrogen and store the samples at −80 °C before use. When needed, thaw an aliquot on ice.

### Ethics statement

The recombinant proteins were produced in accordance with the ethics approval granted by the Ministry of Agriculture of Hungary, Department of Biodiversity and Genetic Engineering, under reference numbers BGMF/81‐9/2024 and BGMF/82‐9/2024. The investigators faithfully adhered to the “Guide for the Care and Use of Laboratory Animals” by the Committee on Care of Laboratory Animal Resources Commission on Life Sciences, National Research Council. Mouse studies (mRNA‐LNP vaccination and ELISA) were conducted under protocols approved by the Institutional Animal Care and Use Committees (IACUC) of the University of Pennsylvania. All animals were housed and cared for according to local, state, and federal policies in an Association for Assessment and Accreditation of Laboratory Animal Care International (AAALAC)‐accredited facility.

### Tips & Tricks

1. We use the SixPack *E. coli* strain because it is specifically designed to efficiently produce rare codon‐containing eukaryotic proteins [14]. Other *E. coli* strains can also be used, such as BL21(DE3) or Rosetta2(DE3), which are suitable for protein expression in the T7 promoter system.
2. LB broth is typically used for producing these antigens, but other types of bacterial media can also be employed. These include terrific broth, super‐broth, or auto‐induction media from various vendors.
3. All chemicals should be of high purity, such as molecular biology or analytical grade.
4. Other high‐capacity IMAC resins, such as Ni‐NTA, cobalt‐based resins such as TALON matrices from various vendors, can also be used. While batch purification is commonly employed, alternative methods like gravity flow affinity columns or prepacked FPLC columns are also effective for purifying these antigens.
5. These antigens are expressed at high levels at 37 °C in 3 h and typically form inclusion bodies. Lowering the expression temperature to 18–22 °C and reducing the IPTG concentration to 0.1–0.2 mm may slightly increase protein solubility, although this can also reduce the overall yield.
6. This protocol is based on using a Branson digital sonifier model 250 with a tapered microtip. Alternative cell disruption methods can also be employed, such as chemical lysis, emulsion flex, or French press.
7. The solubilized antigens can be directly used for IMAC purification, employing standard on‐beads refolding and elution methods, such as gradual renaturation and elution with low pH puffer. However, we have found that refolding by dilution, as detailed in Step III, significantly enhances the solubility, stability, and purity of the final antigens.
8. If you are uncomfortable with decanting, use a pipette, aspirator, or syringe to remove the washing solution from the beads. To avoid losing beads, attach a G25 or G26 needle to the aspirator or syringe.
9. Other types of buffer exchange and/or concentration can also be followed, including tangential flow filtration, dialysis, or size‐exclusion chromatography.

## Conflict of interest

NP is named on patents describing the use of nucleoside‐modified mRNA in lipid nanoparticles as a vaccine platform. He has disclosed those interests fully to the University of Pennsylvania, and he has in place an approved plan for managing any potential conflicts arising from licensing of those patents. NP served on the mRNA strategic advisory board of Sanofi Pasteur in 2022. NP is a member of the Scientific Advisory Board of AldexChem and Bionet‐Asia. ZL held a consulting position at ATGandCo Biotechnology Ltd. at the time the study was conceived. ATGandCo Biotechnology Ltd. was not directly involved in the design or execution of the experiments, nor in the writing of the manuscript.

## Author contributions

EA and ZL performed cloning, protein expression, and purification. HM, RFM, and NP generated mRNA‐LNP vaccines, immunized mice, and performed ELISA. ZL and RFM created the figures. RFM, DYB, NP, and ZL wrote the research protocol and contributed to the funding.

## Data Availability

Data sets presented in the figures and the experimental setups are available upon request. Protein sequences of E140 antigens (Table 1) were retrieved from the UniProt database (https://www.uniprot.org/; UniProt ID: A0A509ADM3 and A0A564ZQP0). pET‐DUET1‐Pb1 and pET‐DUET1‐Pv1 plasmids are available upon request for nonprofit research organizations.
